# Association of IgG1 Antibody Clearance with FcγRIIA Polymorphism and Platelet Count in Infliximab-Treated Patients

**DOI:** 10.3390/ijms22116051

**Published:** 2021-06-03

**Authors:** Gilles Thibault, Gilles Paintaud, Hsueh Cheng Sung, Laurie Lajoie, Edouard Louis, the GETAID, Celine Desvignes, Hervé Watier, Valérie Gouilleux-Gruart, David Ternant

**Affiliations:** 1 EA 7501 GICC, Université de Tours, 37032 Tours, France; gilles.paintaud@univ-tours.fr (G.P.); sophismes@gmail.com (H.C.S.); laurie.lajoie@univ-tours.fr (L.L.); celine.desvignes@univ-tours.fr (C.D.); herve.watier@univ-tours.fr (H.W.); valerie.gouilleux@univ-tours.fr (V.G.-G.); david.ternant@univ-tours.fr (D.T.); 2 Laboratoire d’Immunologie, CHRU de Tours, 37032 Tours, France; 3 Laboratoire de Pharmacologie-Toxicologie, CHRU de Tours, 37044 Tours, France; 4 Department of Gastroenterology, University Hospital, CHU of Liège, 4000 Liège, Belgium; edouard.louis@ulg.ac.be

**Keywords:** FcγRIIA, polymorphism, platelets, Fc–Fc receptor interaction, immunotherapy, monoclonal antibodies, clearance, IgG subclasses

## Abstract

The FcγRIIA/CD32A is mainly expressed on platelets, myeloid and several endothelial cells. Its affinity is considered insufficient for allowing significant binding of monomeric IgG, while its H131R polymorphism (histidine > arginine at position 131) influences affinity for multimeric IgG2. Platelet FcγRIIA has been reported to contribute to IgG-containing immune-complexe clearance. Given our finding that platelet FcγRIIA actually binds monomeric IgG, we investigated the role of platelets and FcγRIIA in IgG antibody elimination. We used pharmacokinetics analysis of infliximab (IgG1) in individuals with controlled Crohn’s disease. The influence of platelet count and FcγRIIA polymorphism was quantified by multivariate linear modelling. The infliximab half-life increased with R allele number (13.2, 14.4 and 15.6 days for HH, HR and RR patients, respectively). It decreased with increasing platelet count in R carriers: from ≈20 days (RR) and ≈17 days (HR) at 150 × 10^9^/L, respectively, to ≈13 days (both HR and RR) at 350 × 10^9^/L. Moreover, a flow cytometry assay showed that infliximab and monomeric IgG1 bound efficiently to platelet FcγRIIA H and R allotypes, whereas panitumumab and IgG2 bound poorly to the latter. We propose that infliximab (and presumably any IgG1 antibody) elimination is partly due to an unappreciated mechanism dependent on binding to platelet FcγRIIA, which is probably tuned by its affinity for IgG2.

## 1. Introduction

The half-life of serum IgG1, IgG2, IgG4 and albumin, which bind to the MHC class I-related neonatal Fc receptor (FcRn) [1], is about 19 to 21 days [2,3], whereas that of IgG3, in which the H435N substitution in the CH3 domain abolishes the affinity for FcRn, is about 5 to 7 days [2,3,4]. This reduced half-life of IgG3 explains why the therapeutic monoclonal antibodies (mAbs) currently in use are based on human IgG1, 2 and 4 structures. Serum proteins, including IgG, are taken up by endothelial cells or haematopoietic cells [5], which express FcRn mainly in intracellular vesicles. Internalization of soluble proteins involves non-specific fluid-phase pinocytosis and/or specific receptor-mediated endocytosis. Following their uptake, proteins traffic into the acidic endosomes, then non-FcRn–binding proteins undergo lysosomal degradation [3,6,7,8].

The high-affinity FcRn–IgG interaction at low pH (≤6.5) within endosomes diverts a fraction of internalized IgG (except IgG3) from degradation: FcRn–IgG complexes escape from the lysosomal pathway and move to the cell surface, where they dissociate at neutral pH, thereby allowing IgG release in the blood and FcRn re-cycling [3,6,7,8,9,10,11]. The site-specific deletion of FcRn to generate mice harboring FcRn-deficient macrophages but not FcRn-deficient dendritic cells or B cells resulted in IgG hypercatabolism, which underlines a pivotal role for FcRn-mediated salvage compensating for the high degradative activities of macrophages in mice [12]. Recently, the lack of a glycine at position 236 in the lower hinge of human IgG2 (as compared with other subclasses) was found associated with increased intracellular catabolism in FcRn-expressing cells [13]. This observation was unexpected in light of the similar serum half-lives of IgG1 and IgG2 [2,3]. The authors assumed that an unknown protective mechanism affecting mainly IgG2 compensated for their decreased FcRn-mediated salvage [13]. Alternatively, it may be postulated that an unknown degradation mechanism affecting preferentially IgG1 might compensate for their increased salvage.

In humans, the primate-specific low-affinity FcγRIIA/CD32A is mainly expressed on platelets, myeloid cells and different types of endothelial cells [14,15,16,17,18,19,20]. Given their relative abundance in the blood and their number of copies on each cell, platelets and neutrophils are the major FcγRIIa expressing populations [21,22]. FcγRIIA is considered a low-affinity receptor, as defined by its ability to bind IgG-containing immune complexes (ICs) but weakly bind monomeric IgG [23,24]. FcγRIIA is encoded by the FCGR2A gene, with allelic dimorphism (rs1801274) resulting in a histidine to arginine substitution at position 131 (H131R) [25,26]. Both FcγRIIA allotypes effectively and similarly bind IgG1-containing ICs or IgG1 dimers, whereas only the FcγRIIA-131H allotype effectively binds those containing IgG2 [25,26,27]. The role of FcγRIIA and the impact of its H131R polymorphism on the catabolism of human IgG or mAbs are still elusive. However, it has been proposed that platelet FcγRIIA, by facilitating the binding of IgG-containing ICs, may provide a mechanism for elimination by professional phagocytes, thus contributing to clearance [28,29]. Given our recent finding that FcγRIIA binds monomeric IgG at physiological concentrations [30], we wondered whether the platelet-dependent clearance mechanism of ICs may extend to monomeric IgG including therapeutic mAbs.

The elimination of IgG is barely evaluable in humans, while pharmacokinetics (PK) studies allow for the evaluation of the elimination of therapeutic mAbs. Free mAbs (i.e., unbound to their target antigen) are eliminated like any IgG via the classical lysosomal degradation mechanism. However, mAb elimination may be increased by the target-mediated clearance (TMC, also called target-mediated drug disposition) mechanism [31,32], in which both IC components (mAb and antigen) are degraded by several mechanisms involving complement or FcγR-expressing cells. Here, we used data from another study designed to identify the individual factors predicting the risk of relapse after discontinuation of infliximab (IFX, a human anti-TNF-α IgG1 mAb) in patients with Crohn’s disease (CD) [33]. These individuals had undergone a period of prolonged remission at inclusion that resulted in reduced TNFα concentration and very low TMC of IFX [33]. The elimination of IFX in these patients may therefore be considered a model of antigen-independent IgG1 mAb elimination, allowing us to study the influence of FcγRIIA and platelets in this process.

## 2. Results

### 2.1. Effect of FcγRIIA Polymorphism and Platelet Count on IFX Pharmacokinetics

Of the 107 participants tested (Table 1) for the FCGR2A-131H/R polymorphism, 31 (29%) and 23 (21.5%) had HH and RR genotypes, respectively, and 53 (49.5%) were heterozygous. These frequencies are similar to those observed in healthy donors [34]. Because their doses and dosing schedule had remained stable for at least 6 months, these participants were assumed to have steady-state IFX concentrations. Our previous PK model allowed us to estimate individual IFX elimination half-lives (t½) with good accuracy [35]. All tested patient characteristics were non-significantly different between *FCGR2A* genotypes, except for IFX t½: the median IFX t½ of all participants was 14.2 days, whereas that for HH, HR and RR participants was 13.2, 14.4 and 15.6 days, respectively. IFX elimination increased and therefore t_½_ decreased (Table 2) with increasing serum C-reactive protein (CRP) level (*p* = 0.0367), as previously described [35].

The fact that CRP level, which is TMC-related [35], was estimated in the model implies that other factors influencing t½ were TMC-independent. IFX elimination also increased with increasing serum IgG1 level (*p* = 0.027) but was not significantly associated with serum IgG2 level and was less associated with IgG1+IgG2 than IgG1 level. IFX elimination decreased with number of *FCGR2A R* alleles (*p* = 0.00003). Finally, it was not significantly associated with platelet count alone but was associated with the interaction between platelet count and *FCGR2A* polymorphism: the t½ decreased with increasing platelet count in R carriers (i.e., HR and RR) (*p* = 0.0004). Therefore, IFX t½ can be estimated using the general linear model: t½ = β_0_ + β_1_.CRP + β_2_.IgG1 + β_3_.F2A + γ_1_.Pl*F2A, where F2A is the number of FCGR2A-R alleles and Pl*F2A is the interaction between platelet count (Pl) and *FCGR2A* polymorphism.

**Figure 1:**
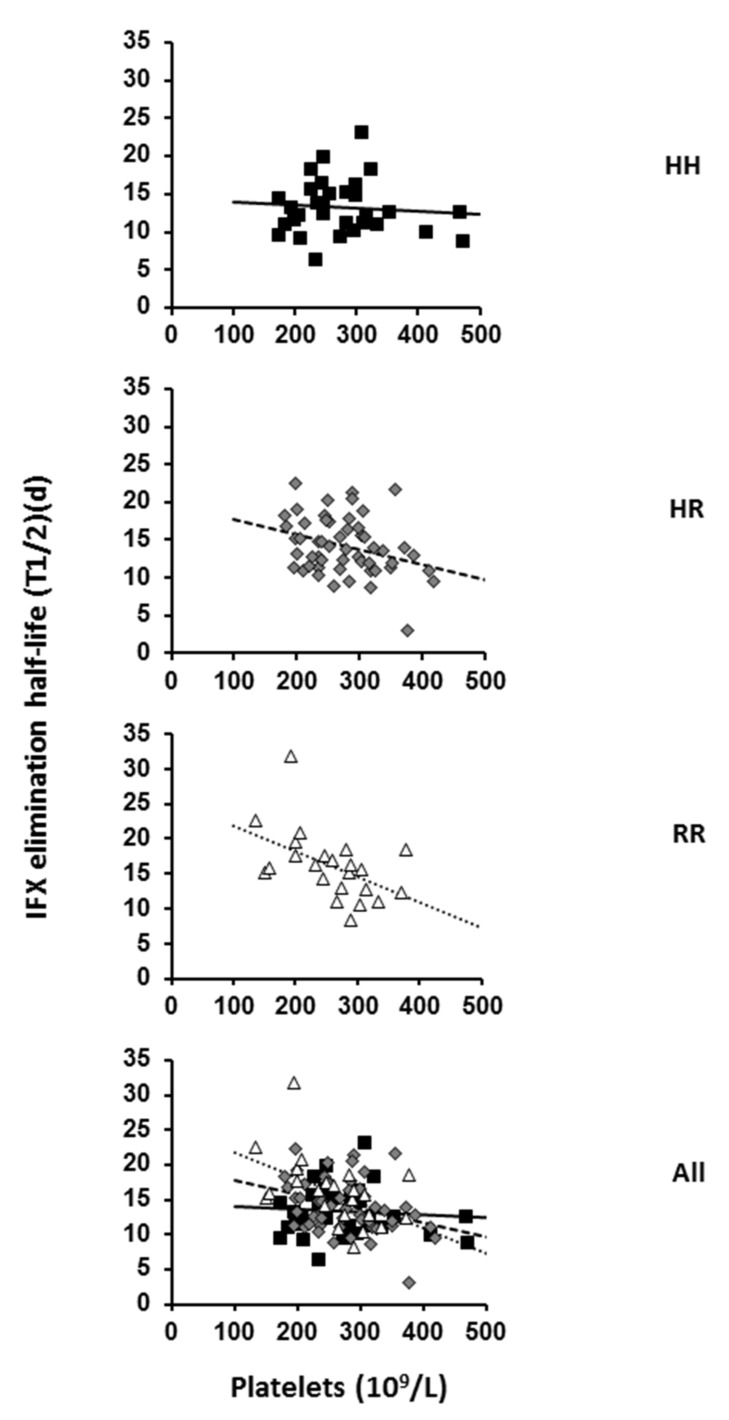
Relationship between IFX elimination and platelet count in *FCGR2A* HH, HR and RR genotyped patients. The IFX t1/2 and baseline platelet counts were evaluated in 31 HH, 53 HR and 23 RR individuals and all participants. Correlation is represented on each graph by the linear regression line (HH: y = −0.004x + 14.237, R^2^ = 0.0069; HR: y = −0.0182x + 19.156, R^2^ = 0.1095 and RR: y = −0.0368x + 25.567 R^2^ = 0.2534).

Using this model, the reference t½ (β_0_) of IFX is 17.3 days for a theoretical HH individual with IgG1 level = 0 g/L, CRP level = 0 g/L and Pl = 0. The t½ of IFX is decreased by 0.46 day with each unit increase in CRP level (mg/L) and by 0.68 day with each unit increase in IgG1 level (g/L). The general linear model includes an interaction term, which allows for the estimation of different linear relationships between IFX t½ and platelet counts for each *FCGR2A* genotype. From this term, t½ of IFX increased by 5.8 days with each R allele (independent of other variables) and decreased with increasing platelet count in *FCGR2A-R* carriers but not HH individuals (*p* < 0.0004). The model-estimated t½ of IFX was decreased by 0.017 and 0.034 days with each unit increase in platelet count (10^9^/L) in HR and RR patients, respectively, independent of other variables. The effect of H131R polymorphism on t½ was substantial at low platelet count (about 20, 17 and 14 days for RR, HR and HH, respectively, at 150 × 10^9^/L) but disappeared at high platelet count (about 13 days whatever the genotype at 350 × 10^9^/L) (Figure 1).

From these results, it can be concluded that the pharmacokinetics of IFX as well as the relationship between IFX elimination and platelet count are associated at least in part with the FcγRIIA expressed on platelets.

### 2.2. Binding of IFX, Panitumumab, or Myeloma IgG1 and IG2 to Platelet FcγRIIA-131H and FcγRIIA-131R

**Figure 2:**
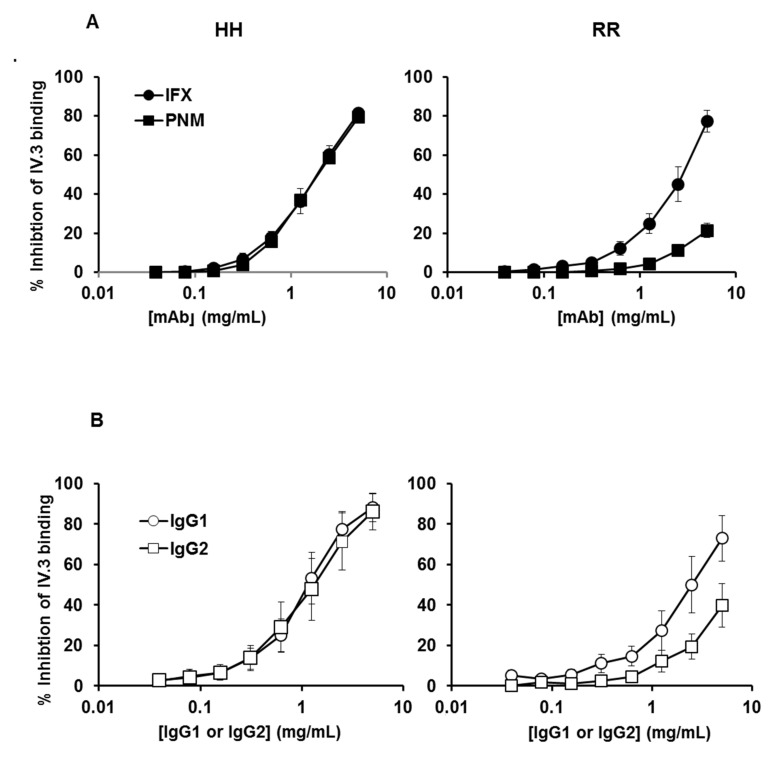
Binding of IFX and PNM or myeloma IgG1 and IgG2 on platelet FcγRIIA according to H131R polymorphism. Inhibition of the anti-FcγRIIA mAb IV.3 binding on washed platelets from HH (**left**) and RR (**right**) donors. The binding of FITC–conjugated IV.3 on platelets in the presence of increasing concentrations of monomeric mAbs (**A**) or myeloma IgG (**B**) was assessed by flow cytometry. Percentages of inhibition of IV.3 binding were calculated as described in “Materials and Methods”. Results are means ± error from three flow cytometry analysis of three different experiments (i.e., performed with platelets from three different pairs of HH and RR donors).

The affinity of FcγRIIA is usually considered insufficient for allowing significant binding of monomeric IgG [23,24], although previous studies have shown that monomeric IgG is a functional antagonist of FcγRII [36,37]. To challenge this assertion, our recent flow cytometry experiment evaluated the ability of polyclonal IgG or cetuximab, a chimeric anti-EGFR IgG1 mAb, or panitumumab (PNM), a fully human IgG2 anti-EGFR mAb, to inhibit the binding of FITC-conjugated IV.3 mAb (whose epitope is located within the IgG binding site of FcγRIIA) on washed platelets from HH and RR individuals [30]. A direct method, using fluorochrome-labelled secondary antibodies (anti-kappa chain), was achievable. However, such a method requires that the secondary antibody binds identically to each ligand (whatever its structure), while our indirect method avoids this difficulty. In the present study, we used this approach and compared the binding of (monomeric) IFX and PNM. Both IFX and PNM from 1 to 10 mg/mL greatly inhibited the binding of IV.3 on platelets from HH donors (Figure 2A left).

The inhibition profiles with the two mAbs on platelets from HH donors were strictly similar: a concentration of about 1.5 mg/mL for each mAb was sufficient to achieve 50% inhibition. The profiles were different when platelets from RR donors were used (Figure 2A right): as compared with IFX, PNM less potently inhibited the binding of IV.3 mAb. An IFX concentration of about 2.5 mg/mL was required to achieve about 50% inhibition, whereas a similar PNM level led to about 10% inhibition. These findings confirm the results obtained previously with cetuximab and PNM [30].

We then verified that “natural” (i.e., non-bioengineered) human IgG1 and IgG2 had the same properties as mAbs. Therefore, we used the same assay to compare the affinity of human myeloma IgG1 and IgG2 proteins. Results obtained with myeloma proteins were similar to those obtained with the therapeutic mAbs: a concentration of about 1.25 mg/mL for each myeloma protein was sufficient to achieve 50% inhibition on HH platelets, whereas a concentration of about 2.5 mg/mL of IgG1 and IgG2 resulted in about 50% and 20% inhibition, respectively on RR platelets (Figure 2B). These findings show that: (1) both allotypes are able to bind monomeric IgG or mAbs; (2) the plateletFcγRIIA-131H allotype effectively and similarly bound IFX, PNM, IgG1 and IgG2 myeloma proteins (at a level corresponding to those of circulating endogenous IgG1 and IgG2; i.e., 4 to 9 mg/mL and 2.5 to 7 mg/mL, respectively, in healthy adults); (3) the platelet FcγRIIA-131R efficiently bound IFX and the IgG1 myeloma protein, although slightly less than the FcγRIIA-131H; and (4) the platelet FcγRIIA-131H poorly bound PNM or IgG2 myeloma protein.

## 3. Discussion

The goal of this study was to investigate the involvement of FcγRIIA and platelets on eliminating IgG1 mAbs in individuals receiving IFX for CD as a model. The elimination of IFX decreased with number of R alleles (t½ rank order HH < HR < RR). Importantly, IFX elimination was inversely related to platelet count in RR and HR individuals. In addition, IFX and myeloma monomeric IgG1 bound efficiently to platelet FcγRIIA. Thus, we propose that the elimination of IFX and probably that of all IgG1 therapeutic mAbs results in part from an unappreciated elimination mechanism involving the binding of these molecules to platelet FcγRIIA.

Two main mechanisms have been proposed to explain the elimination of mAbs. The first is TMC, in which the mAb is “consumed” after IC formation with the antigen (TNF-α in the case of IFX) [31,32]. Because in individuals with substantial inflammation, the production of TNF-α leads to increased CRP level, serum CRP level may be considered an indirect marker of TNF-α concentration [35]. Therefore, the relationship between CRP level and t½ observed previously and confirmed here [35] may be explained by an increase in IFX target-mediated elimination rate. However, the low impact of CRP level in our multivariate model agrees with the patients having controlled disease at inclusion (CDAI ≤ 150). Therefore, TMC was weakly involved in the elimination of IFX in our study, and IFX elimination may be considered an accurate marker of antigen-independent IgG1 mAb elimination in our patients.

The second mechanism, related to the IgG structure of mAbs, is the FcRn-regulated antigen-independent lysosomal degradation mediated by IgG-endocytosing cells, which are mainly endothelial and hematopoïetic cells [5]. The substantial effect of platelet count and FCGR2A polymorphism on IFX t½ strongly suggests that an additional mechanism due to binding of the mAb to platelets via FcγRIIA is involved in the antigen-independent clearance of IFX. Platelets do not express FcRn, and its encoding gene FCGRT is on chromosome 19, which excludes linkage disequilibrium with FCGR2A (on chromosome 1). Therefore, the platelet-dependent mechanism is not counteracted by FcRn salvage in platelets. The IFX t½ substantially decreased with increased platelet count in HR and RR individuals, so the contribution of platelet-mediated elimination became highly significant at high platelet counts in these individuals. The accelerated elimination of IFX observed at high platelet count agrees with the high turnover of platelets; because of their 7 to 10-day lifespan, 10^11^ platelets (10–15% of the circulating pool) are renewed every days [38]. It is of note that the IFX t½ did not decrease significantly with increased platelet count in HH individuals. Monomeric monoclonal IgG1 and IgG2 as well as the IgG1 IFX and IgG2 PNM mAbs bound similarly to the FcγRIIA-131H allotype. Furthermore, these bindings were similar to that of polyclonal IgG, which we reported previously [30]. In addition, FcγRIIA-131R, which had moderately lower affinity for IgG1 than FcγRIIA-131H, weakly bound PNM and monomeric IgG2. The ability of the low-affinity FcγRIIA to actually bind the Fc portion of monomeric IgG at physiologic concentrations that we report agrees with previous reports [36,37,39]. It follows from these results that at least a fraction of platelet FcγRIIA receptors is occupied by IgG in the in vivo situation. Due to its IgG1 structure, IFX competes with endogenous IgG for the binding to platelet FcγRIIA. However, given that the FcγRIIA-131H allotype binds similarly IFX, IgG1 and IgG2 (Figure 2), IFX competes with both subclasses (i.e., about 90–95% of total plasma IgG) for binding to platelets expressing this allotype. The level of IFX bound to platelet FcγRIIA-131H depends on the ratio between serum concentration of IFX and that of IgG1+IgG2. By contrast, given that the FcγRIIA-131R allotype binds poorly IgG2 compared with IFX and IgG1 (Figure 2), IFX competes mainly with IgG1 (i.e., about 55% of total plasma IgG) for binding to platelets expressing this allotype. The level of IFX bound to platelet FcγRIIA-131R depends primarily on the ratio between the serum concentration of IFX and that of IgG1. Accordingly, the percentage of FcγRIIA-131R receptors occupied by IFX is higher than that of FcγRIIA-131H receptors at a given serum concentration of IFX. Therefore, the relative proportion of platelet-bound IFX increases with the number of FcγRIIA-131R receptors expressed on each platelets (conditioned by the number of R allele), which explains why the elimination of IFX increased substantially with the platelet count in RR individuals, whereas it increased less markedly in HR (two times less vs. RR) and very weakly if not at all in HH patients. In addition, we observed that the H131R polymorphism had marginal impact on IFX elimination at high platelet count (Figure 2B). Conversely, elimination decreased substantially with number of R alleles at low platelet count, i.e., when elimination was mainly if not exclusively mediated by IgG-endocytosing cells. The results show that FcγRIIA plays also a role in the lysosomal-dependent mechanism. It strongly suggests that the R to H substitution at position 131 is associated with a shift in functionality from FcRn salvage toward lysosomal degradation in FcγRIIA-expressing cells. This is consistent with previous studies showing that FcγRIIA and FcRn are co-expressed on several cells involved in IgG catabolism, such as monocytes/macrophages (and probably neutrophils, which represent the other major FcγRIIA-expressing population with platelets) or liver, heart or dermal endothelial cells, and may functionally cooperate [17,18,19,20,40,41,42]. We propose that the catabolism of IFX and probably that of all IgG1 therapeutic mAbs (and polyclonal antibodies) in the absence of antigen binding results from two additive mechanisms whose relative part depends on the platelet count and FcγRIIA polymorphism: the classical FcRn-regulated lysosomal-dependent mechanism occurs after endocytosis by myeloid and endothelial cells and the FcRn-non-regulated one occurs after binding to platelet FcγRIIA. In addition, we propose that the latter is tuned by the affinity of IgG2 for the FcγRIIA allotypes.

Our findings may explain at least in part the unexpected observation that IgG1 and G236 missing IgG2 have similar half-lives despite increased intracellular catabolism of IgG2 in FcRn-expressing cells [13]. Indeed, IgG1 will be more efficiently eliminated by the platelet-dependent mechanism in individuals expressing at least one R allele (i.e., HR and RR), which represents about 75% of individuals, compensating for higher FcRn-mediated salvage. Finally, IgG3, whose short half-life has been related to the H435 mutation leading to low affinity for FcRn and poor salvage [4], has high affinity for FcγRIIA (both allotypes) [24], favoring strong platelet-dependent elimination. Conversely, the long half-life of IgG4 agrees with the substantial affinity for FcRn and low affinity for FcγRIIA limiting both elimination mechanisms.

In summary, our study supports the existence of an unanticipated mechanism of IgG1 (and possibly IgG2) mAb elimination resulting from binding to FcγRIIA expressed on platelets. Although this platelet-dependent elimination mechanism requires further investigation, platelet-bound IFX or IgG1 (and probably PNM or IgG2 in the case of HH carriers) may be catabolized similarly to IgG-containing IC after platelet internalization and clearance by professional phagocytes [28,29]. Despite the uncertainty of the mechanism, which may be phagocytosis or endocytosis, our results show that the catabolism of therapeutic IgG1 mAbs is associated with the FcγRIIA polymorphism and the platelet count, which suggests that these parameters could impact the clinical responses to these agents.

## 4. Materials and Methods

### 4.1. Patients and Biological Analyses

The present work is an ancillary analysis of a prospective multicenter cohort study (ClinicalTrials.gov NCT00571337 (accessed on 13 May 2021)) whose results are described in detail elsewhere [33]. The study protocol and documents were approved by the Ethics Committee of Saint-Louis Hospital in Paris (26 May 2005). All patients gave their written informed consent before screening. The princeps study was designed to assess the risk of relapse of individuals with CD in remission after IFX discontinuation. Included CD patients (1) had received IFX and an antimetabolite agent (azathioprine, 6-mercaptopurine or methotrexate); (2) had a prospective Crohn’s Disease Activity Index (CDAI) ≤ 150; (3) had stable disease for at least 3 months and were in corticosteroid-free remission during the 6 months before inclusion; and (4) had at least 1 year of scheduled IFX infusions and at least two infusions administered during the previous 6 months. Participants were followed for 30 months or until relapse, study withdrawal or study closing date.

Platelet counts and serum IgG, IgG1 and IgG2 levels were measured at baseline by routine procedures, and IFX serum level was measured at baseline and at each scheduled visit or at the time of relapse by ELISA [43]. Genotyping of FCGR2A was performed as described [30]. A total of 115 participants were analyzed in the princeps study. In the present study, we analyzed data for participants with available *FCGR2A* genotype information (*n* = 107).

### 4.2. Pharmacokinetics Analysis

The PK analysis of IFX is described in detail elsewhere [35]. In the previous study, IFX concentrations were described using a one-compartment population model. From this model, individual values of IFX t½ were computed. This index reflects the elimination rate of the studied drug: the longer the t½, the lower the elimination rate.

### 4.3. Antibodies

The mAb for anti-CD32 (clone IV.3) was from Stemcell Technologies (Grenoble, France). Myeloma IgG1 and IgG2 were from Sigma-Aldrich (Saint-Quentin Fallavier, France). IFX, a chimeric mouse-human IgG1 anti-TNF antibody, and panitumumab (PNM), a human IgG2 anti-epidermal growth factor receptor (EGFR) antibody, were from Schering-Plough and Amgen, respectively.

### 4.4. Washed Platelet Preparation

Whole blood from healthy donors was collected in plastic tubes containing sodium citrate anticoagulant solution (BD Vacutainer) supplemented with 1 µg/mL prostaglandin E1 (Sigma). The mixture was centrifuged at 150 g for 15 min at room temperature. The supernatant (platelet-rich plasma) was collected in a clean tube and centrifuged at 2500 g for 15 min at room temperature. The supernatant was discarded, and the platelet pellet was carefully resuspended in a washing solution consisting of 103 mM NaCl, 36 mM citrate acide, 5 mM glucose, 5 mM KCL, 2 mM CaCl2 and 1 mM MgCl2 (pH 6.5). The final cell suspension was centrifuged at 2500 g for 15 min at room temperature. The platelets were resuspended in PBS containing 1% BSA (Sigma) to a final volume sufficient for a platelet suspension containing 4 × 10^8^ platelets/mL. Platelet counts were obtained by using Z2 COULTER COUNTER (Beckman Coulter).

### 4.5. Flow Cytometry Assay of mAb or Myeloma IgG Binding to Platelet FcγRIIA

IV.3 is a mouse IgG2b mAb that blocks the binding of IgG to FcγRIIA, so its epitope is within or near the Fc binding site. Therefore, we evaluated the ability of monomeric IgG to inhibit the binding of FITC-conjugated IV.3 to FcγRIIA on platelets from HH or RR donors [29], using a procedure similar to an assay we had previously developed to evaluate IgG–FcγIIIA interaction [44]. Briefly, platelets (5 × 10^4^ in 10 μL) were incubated with various concentrations of IFX or PNM or myeloma IgG1 or IgG2 for 30 min at room temperature, then with 10 μL FITC-conjugated IV.3 (1 μg/mL, 30 min, room temperature) and analyzed by flow cytometry after adding 300 μL PBS containing 1% BSA. Results are expressed as percentage inhibition of IV.3 binding: (%IV.3-positive platelets in the absence of IFX, PNM, IgG1 or IgG2—%IV.3-positive platelets in the presence of IFX, PNM, IgG1 or IgG2) × 100/(%IV.3-positive platelets in the absence of IFX, PNM, IgG1 or IgG2).

### 4.6. Statistical Analysis

Patient characteristics were compared between *FCGR2A* genotypes using Kruskall–Wallis ANOVA nonparametric test for continuous variables, or chi-square test for proportion data. All tests were bilateral with alpha risk fixed at 5%.

The association of individual factors with IFX t½, computed from individual pharmacokinetic parameters, was quantified by multivariate linear modelling. The dependent variable was IFX t½. Independent variables were CRP, IgG, IgG1 and IgG2 concentrations; platelet count; and FCGR2A (HH, HR and RR) genotypes. The association with *FCGR2A* polymorphism and the platelet count, as well as first-order interaction between these two factors (the interaction term allows different slopes of t½ vs. Pl for each *FCGR2A* genotype), was tested in the ANCOVA model by using Fisher F-test. Analyses were performed with R v3.2.3 (Vienna, Austria). A *p*-value < 0.05 was considered statistically significant.

## Figures and Tables

**Table 1:** Patient characteristics.

		*FCGR2A* Genotype		
Characteristics	Total	HH	HR	RR
Number of patients ^1^	107	31	53	23
Sex, women ^†^ [n (%)]	47 (42)	13 (42)	22 (42)	12 (52)
Age ^†^, years	31 (25–39)	29 (25–35)	33 (27–43)	30 (26–37)
Body weight ^†^, kg	67 (57–75)	68 (63–79)	62 (56–75)	72 (58–78)
Surgery ^†^ [n (%)]	22 (21)	5 (16)	14 (26)	3 (13)
CDAI ^†^	36 (17–60)	24 (6–54)	36 (21–61)	40 (17–53)
CDEIS ^†^	0.7 (0.0–3.0)	0.8 (0.0–3.0)	0.4 (0.0–2.0)	1.8 (0.1–3.4)
HsCRP ^†^, mg/L	2.2 (0.8–4.8)	2.6 (1.5–4.7)	1.7 (0.7–4.2)	2.0 (0.9–5.2)
IgG ^†^, mg/mL (range)	12.6 (6.6–17.8)	11.9 (8.6–17.4)	13.0 (6.6–17.8)	12.6 (8–16.7)
IgG1 ^†^, mg/mL (range)	6.0 (3.3–12.7)	5.6 (3.6–8.6)	6.2 (3.3–12.2)	6.0 (3.6–9.8)
IgG2 ^†^, mg/mL (range)	5.0 (1.8–8.7)	4.9 (2.5–8.2)	5.0 (1.8–8.7)	4.9 (2.9–8.7)
Platelets ^†^, 10^9^/L (range)	272 (135–471)	255 (135–379)	274 (181–420)	266 (174–471)
Infliximab * t½, d	14.2 (12.2–16.4)	13.2 (11.3–16.0)	14.4 (12.4–16.3)	15.6 (13.6–17.3)

^1^ Data are median (interquartile range) unless specified otherwise. CDAI, Crohn’s Disease Activity Index, CDEIS, Crohn’s Disease Endoscopic Index of Severity, HsCRP High-sensitivity C-Reactive Protein. ^†^ Non-significantly different (between *FCGR2A* genotypes); * *p*-value = 0.018 (between *FCGR2A* genotypes).

**Table 2:** Factors associated with infliximab (IFX) elimination.

Factor ^1^	Parameter	Value (d)	*p*-Value
(Intercept)	β_0_	17.3714	<0.00001
CRP serum concentration (CRP)	β_1_	−0.4646	0.0367
IgG1 serum concentration (IgG1)	β_2_	−0.6766	0.0269
*FCGR2A* R alleles (F2A)	β_3_	5.7478	0.00003
Interaction between platelet count and *FCGR2A* polymorphism (Pl*F2A)	γ_1_	−0.0170	0.0004

^1^ The effect of individual factors on t½, estimated by population post-hoc analysis, was quantified by multivariate linear modelling. The effect of FCGR2A and platelet count as well as first-order interactions between these two factors were tested in the ANCOVA model, using Fisher *F*-test. Coefficient of determination (R2) of the final model was 27.04%.

## Data Availability

Not Applicable.
